# Why do women choose private over public facilities for family planning services? A qualitative study of post-partum women in an informal urban settlement in Kenya

**DOI:** 10.1186/s12913-015-0997-7

**Published:** 2015-08-20

**Authors:** Sirina R. Keesara, Pamela A. Juma, Cynthia C. Harper

**Affiliations:** Bixby Center for Global Reproductive Health, Department of Obstetrics, Gynecology & Reproductive Sciences, University of California, San Francisco, USA; African Population Health Research Center, Nairobi, Kenya

## Abstract

**Background:**

Nearly 40 % of women in developing countries seek contraceptives services from the private sector. However, the reasons that contraceptive clients choose private or public providers are not well studied.

**Methods:**

We conducted six focus groups discussions and 51 in-depth interviews with postpartum women (*n* = 61) to explore decision-making about contraceptive use after delivery, including facility choice.

**Results:**

When seeking contraceptive services, women in this study preferred private over public facilities due to convenience and timeliness of services. Women avoided public facilities due to long waits and disrespectful providers. Study participants reported, however, that they felt more confident about the technical medical quality in public facilities than in private, and believed that private providers prioritized profit over safe medical practice. Women reported that public facilities offered comprehensive counseling and chose these facilities when they needed contraceptive decision-support. Provision of comprehensive counseling and screening, including side effects counseling and management, determined perception of quality.

**Conclusion:**

Women believed private providers offered the advantages of convenience, efficiency and privacy, though they did not consistently offer high-quality care. Quality-improvement of contraceptive care at private facilities could include technical standardization and accreditation. Development of support and training for side effect management may be an important intervention to improve perceived quality of care.

## Background

In prior decades, moving to an urban setting in Africa meant growing richer and having better access to institutional services. However, recent migration studies in Sub-Saharan Africa show that in the next decade, nearly 70 % of the urban population will live in poverty and settle in low income, informal settlements without designated public services [1]. When separating the health indictors of this new urban population from those of the urban population as a whole, prominent disparities emerge. For example, people living in the slums areas of Nairobi have higher rates of unintended pregnancy and fertility than those living in non-slum areas [2]. Contraceptive prevalence rate (CPR) in the slum areas of Nairobi is around 45 % while the CPR in non-slum areas is 50 % [2]. In urban areas of Kenya, the injectable contraceptive is the most commonly used method [3].

As contraceptive prevalence rates increase in Sub-Saharan Africa, private venues are becoming more popular [4–6]. In Kenya, more than 40 % of contraceptive users obtain contraceptives at private hospitals, pharmacies, and dispensaries [7]. Private and unlicensed facilities dominate the healthcare landscape in poor neighborhoods of Nairobi. Of 125 healthcare facilities surveyed in a poor urban area of Nairobi, 84 % were private facilities, and only 47 % of these had licenses [8]. Poorly maintained road networks and relatively high cost of transport hinder access to public healthcare facilities, which have cheaper and more standardized services [9–11].

Social franchise networks and public-private partnerships have strengthened the ability of private facilities to provide high-quality family planning services [12, 13]. Individual and household surveys in urban areas have demonstrated higher satisfaction in private healthcare facilities than in public healthcare facilities [14, 15]. Data from private family planning providers in Ghana, Tanzania and Kenya show that respectful treatment, shorter wait times, and met expectations correlates with higher client satisfaction [16]. Higher satisfaction ratings and willingness to pay may indicate an overall preference for private facilities [17].

Few studies detail perceptions of family planning services offered at different facilities in these communities. Qualitative data can provide insight into reasons that women choose private sector family planning services. This qualitative study describes women’s expectations and experiences when seeking contraceptive care from private and public facilities in Nairobi.

## Methods

From December 2013 to April 2014, we conducted six focus group discussions (FGD) and 51 in-depth interviews (IDI) to learn about barriers to use of postpartum contraception in Mathare Valley informal settlement in Nairobi, Kenya. FDGs were chosen to elicit norms of behavior and social expectations, and IDIs were chosen to explore individual experiences, choices and perception of family planning services. The quality of services in private and public facilities emerged as a secondary outcome within the focus groups and was explored in individual interviews.

The Mathare Valley informal settlement houses between 80,000 and 188,000 Kenyans. The population of Mathare Valley has an average monthly income of 10,000 Kenyan Shillings (KES), around $117 United States Dollars (USD) [18]. Mathare North Health Center (MNHC) is a public hospital located in a western sub-village (population between 18,000 and 30,000) of the Mathare Valley and provides outpatient and low-risk inpatient maternal and child health services [18].

There are few registries of populations living in informal settlements, so women were recruited through healthcare-related networks within the settlement. A study facilitator informed women about the study in waiting rooms of Mathare North Health Center. Referrals from community health workers were included to bring perspectives from women who may not use public facilities for reasons such as mistrust or mistreatment. The facilitator was female, native speaker of Swahili, university-educated, and fluent in English. The facilitator and community health workers collected contact information from women who were interested in the study and the facilitator called the potential participants within a week. If the potential participant chose to enroll in the study, the facilitator arranged a meeting for a focus group or interview in a convenient location for the participant. All participants were over 18 years of age.

The focus groups were divided into three categories: women with babies under 6 weeks, women with babies 6 weeks- 6 months old, and women with babies 6 months- 1 year old. In these focus groups, the facilitator used a question guide to lead discussions about use of postpartum contraception including perceptions of family planning services at private and public facilities. The focus groups ranged from eight to twelve postpartum women and were conducted in a private room of a local community center and lasted between 1–2 hours. Women were remunerated for their travel with 100 KES (~ $1.2USD) and for their time and with a small gift (baby oil).

The emergent themes from the focus groups led to revision of the question guide for individual interviews, which probed further into individual use and choice of private or public facilities for family planning services. We conducted semi-structured individual interviews with 30 postpartum women who were not a part of the focus groups. The interviews asked questions about previous use of contraception, intention to use contraception after delivery, and preference for service location. A second set of interviews was conducted approximately 3 months after delivery with 20 women to assess actual use of contraception, reveal barriers to use, and explore perception of quality of family planning services. The interviewer was the same as the focus-group facilitator. To minimize travel for the participants, the interviews were conducted in a private room of the women’s homes or a private interview room at the hospital after they had attended clinic.

The FGDs and IDIs were audiotaped, transcribed verbatim in Swahili and translated into English. An additional translator checked the English transcripts for accuracy. The transcription and translation were done concurrently with data collection. After transcription, audiotapes were destroyed and the transcriptions were de-identified. A participant enrollment log was the only document connecting the participant characteristics to the transcriptions.

The focus groups were reviewed and were analyzed thematically with a deductive process, focusing on themes about use of private and pubic facilities. Two of the investigators (SK and PA) reviewed and analyzed data from the six FGDs and three IDIs. The codes were discussed and revised after review of the IDIs. Then, SK coded the remainder of the interviews using Atlas.ti. Quotes and example stories were chosen to highlight common opinions and behaviors.

The study was conducted with ethical approval from University of California, San Francisco’s Committee on Human Research and Kenya Medical Research Institute’s Ethical Review Committee. Written informed consent was obtained from each participant before interviews and focus groups.

## Results

A total of 91 women participated in the study with 61 focus group participants and 30 in-depth interview participants. Participant characteristics, only available in individual interviews, are shown in Table 1. Ages ranged from 19 to 38 years, but most were under age 35 years. Most had four or fewer children and had 12 years or less of education.

**Table 1:** Participant Age, Education, and Parity of In-depth interview participants (*N* = 30)

Age	
19–24	12
25–29	7
30–34	8
35+	3
Education (years)	
<8	11
8–12	17
12+	2
Parity	
0	0
1–2	12
3–4	17
5+	1

### Easy access, convenience, and respect for client in private care settings

Focus group discussions revealed that most women sought care at private facilities for convenience. Due to an abundance of facilities, women were able to access a private facility easily and experienced little to no wait times at these venues. Even though family planning services were free at the public hospitals, one woman explained that she was willing to pay for contraception at private facilities to avoid waiting in long lines:

There are many private clinics around here that some women prefer to go to because if you go to Mathare North dispensary [public facility] you will have to queue for long. Some women prefer to pay 100 KES at the private clinic and be over with it (Focus Group Participant).

In the individual interviews, women described the convenience of private facilities in more detail. Women reported that private facilities offered long and convenient service hours that accommodated women’s busy schedules. One woman explained that public facilities often closed before they attended to everyone:

I can go [to the private hospital] at anytime. At public health facilities they take long to give service. Some people would wait and even give up and go back home, or some don’t even feel like going there because you would go in the morning and leave at 1:00… So some opted to go to private health facilities because if you wanted an injection you would just walk there and pay then receive the injection, and walk out, and they don’t take a lot of your time and you can go and do other things. (Age 32, 3 children).

Women explained that choosing private facilities or chemists (pharmacies) assured them of medication availability. Some women said that they had wasted time waiting at the public facilities for free services, only to find that their preferred method was not available. One woman began to obtain her contraception at a private facility when she found that public facilities did not stock all methods consistently:

I have only used the injection and pills. I used to buy the pills at the chemist [pharmacist] so I didn’t have to come to the hospital. The first time I started using pills, when I went to the hospital, they didn’t have pills and I had to buy from the chemist so I continued buying from the chemist. (Age 38, 4 children)

Women explained that workers at private facilities always provided whichever method was requested. One woman complained that nurses at the public facility prevented her from switching to the injectable contraceptive, so she went to a private facility where they administered her desired method:

I used pills after my first delivery but within the first two days I really got sickly and I stopped using them. I threw them away because I had headaches, I didn’t feel like doing anything. I went to the [public] hospital they told me that I should perservere and finish the dose. I got upset and I threw them away and went to the private clinic and went for the injection. (Age 34, 2 children)

Another woman explained that she chose a private facility because she wanted to bypass obstructive processes that she foresaw at the public facility. She had planned to obtain the contraceptive implant at a public facility during her six-week postpartum visit. However, when she received her period four weeks after delivery, she opted for a private facility:

…When my periods came [at 4 weeks], I felt like it was an emergency, and I didn’t want to waste more time because, like I mentioned, these men are unpredictable and they might demand for it [sex] at anytime. I had planned on going for the [public] clinic, but when my menses came I asked a friend if they will allow me to take up family planning at the clinic [early] and she told me that they cannot accept. That is why I went for the method at a private health facility. (Age 27, 3 children)

Respectful treatment was an added benefit of private facilities. Women believed that private facilities treated their customers with care and attention compared to public facilities where participants experienced verbal harassment, inattention, and rudeness. Respectful behavior included answering questions kindly and allowing sufficient time for each client. One woman described how rude behavior at public facilities drove clients to private clinics:

For instance if I was using a method and it was not working for me, then I come back to the hospital and the nurse starts yelling at me like, “You woman, don’t be foolish.” You know such things are making many women go to the private facilities because when you go there people respect you. They should respect us and address us like adults and not insult us. (Focus group participant)

Finally, women said they used private facilities when they required more confidentiality. One woman related a story of a friend who chose to receive family planning at a private facility to hide her use from her husband:

Her husband didn’t want her to take family planning but he was not giving her any valid reasons why she shouldn’t take that up, so she just went privately. It was difficult, but she went to a chemist so that she can be able to go when the husband is not around. (Age 24, 2 children)

### Perceived poor-quality technical medical services in private care settings

While efficiency and client-centered care were attractive features of private facilities, women believed that technical quality and patient safety were compromised. The majority of the women in the study recommended attending public facilities for standardized and thorough medical treatment. Focus groups participants noted that the private facilities prioritized profit over providing safe medical treatment. While some women mentioned that private providers at non-governmental organization (NGOs) answered questions fully, most women said that private most facilities did not provide counseling or decision-support when administering a method:

In private clinics it’s about money. They don’t have time to counsel you. They don’t offer good services. By the time you go to the clinic, you have a method in mind, so they just administer. (Focus Group Participant).

In individual interviews, women elaborated on their perceptions of the deficiencies in private facilities, which included questionable medications, poor eligibility screening, poorly qualified staff, and poor quality counseling. Women recognized that choosing private facilities for medical services meant sacrificing thorough eligibility screening. This woman explained her worry that private facilities did not ask questions or run tests before administering a method:

When you visit the private health facilities, they will just ask you what method you want and they won’t offer counseling services. They will not even ask how old the baby is, they will give you what you went for. They will just ask you when you had your last menses and go ahead and maybe you are pregnant they don’t run tests, they will just inject you. (Age 23, 2 children)

Other women were concerned about the competency of private facility providers. This woman explained her concerns about private providers and her preference for well-qualified public providers:

In public facilities the doctors are qualified but in private facilities it could be a quack, or the doctor might be qualified, but he could be using his wife to assist him, but the wife is not qualified. But in public facilities you always find qualified staff from the doctor to all the other employees. So their services are genuine and you don’t get scared when they are attending to you. (Age 34, 4 children)

While it was expected that private facilities would provide a consistent stock of contraceptive supplies, women worried that these facilities administered fraudulent and expired medications to unaware clients. A few women stated that private facilities were more likely to stock expired contraceptives because their inventory exceeded their client flow. This woman attributed two incidences of failed contraception to fraudulent medication provided at private facilities:

Some women are injected with water and you keep feeling safe that you have used family planning while you are unsafe and you eventually you end up pregnant. That has happened to two women. One had gone to the chemist and another one at a private facility. They ask you for money but they don’t render the services. (Age 23, 1 child)

Women recognized the advantages and disadvantages of private and public facilities, and chose according to their immediate needs. For example, one woman weighed the advantage of the fast services at private facilities against the risk of receiving expired drugs. She warned women to diligently check expiration dates of medications they received at private facilities, but ultimately recommended attending public facilities where they would receive genuine medications and high quality medical treatment. Because of the concern for poor quality medical treatment at private facilities, some women said that they preferred to endure long waits at public facilities:

After that story I had heard from those women about private hospitals, I was reluctant to do it [go to private providers] because maybe their medication for family planning is also expired, I was comparing them to the chemists because they are all private businesses. And so I decided to be patient and go to the Government clinics (Age 25, 3 children)

Furthermore, woman in the individual interviews said they preferred public facilities when they needed more decision-making support or guidance for initial selection of a contraceptive method. This woman explained her decision to seek services at public facilities to decide about her method of contraception after delivery:

I got more information from the [Nairobi] City Council hospitals. Private [hospitals] don’t have time for such talk [counseling about family planning]. They are more concerned about their time. So after delivery, regardless of where I have delivered I visit [Nairobi] City Council clinics and they provide training on that. (Age 34, 3 children)

Women recognized questionable quality of medical care at private facilities, which led some to choose public hospitals.

### “High-quality” means thorough counseling

When asked to define high-quality services, overwhelmingly, women described thorough counseling and screening procedures. Scant testing and counseling at private clinics instilled worry about being given an inappropriate method. Many believed that public providers used a “blood test” to screen out methods that would cause side effects. One woman explained her worries about private facility screening methods:

When you walk to a private clinic, you will tell them that you need an injection and when you walk there asking for an injection that is what you will be given. …They don’t do any tests to establish whether you should have used the pills or coil or Norplant and then you end up developing side effects. (Focus Group Participant)

Because of concern for side effects, almost every woman described an ideal family planning visit as one with ample counseling about side effects and support from the provider to choose a method that minimized side effects:

A good family planning visit is whereby when you enter the room you are counseled first, then you get to choose one and she tells you the side effects and she recommends what you should use suiting your body and not just allowing you to go for a method you had already decided to use even if it’s not good. (Age 21, 1 child)

Minimization of side effects was one of the highest priorities when choosing a contraceptive method. While public facilities were able to provide a broad overview of side effects, they were not able to provide individualized attention. Due to crowded facilities in public healthcare settings, some women were not given the opportunity to address problems with their current method. One woman described her disappointment about not receiving adequate counseling from a public facility when she returned with irregular vaginal bleeding:

I expected her to counsel me more about family planning, but the first thing she did when she met us was whine that we were late. She only asked me what method I wanted and I told her the injection and that was it. (Age 23, 1 child)

The poor individual attention at public facilities disappointed many women when they encountered side effects. Interestingly, one woman believed that NGOs provided higher quality care because they were able to manage her side effects and provide extensive individual attention. Because her point of reference had shifted, she did not believe that public facilities provided high quality care.

Overall, description of high quality care revolved around individualized care and minimization of side effects.

## Discussion

These results displayed women’s experiences in public and private facilities and elucidated the reasons that determined facility choice. Findings of this study suggested that women in the informal settlement in Nairobi valued private facilities for timely services and attended these outlets when they had already decided upon a contraceptive method. According to previous studies, decreased waiting times and administration of preferred method are two important components of quality of care that lead to satisfaction with services and promote continued contraceptive use [12, 13, 15]. A qualitative study of private maternal healthcare facilities in Kenya show that proximity of a health facility to home increased perceived quality of services [10]. With limited mobility and numerous household responsibilities, women who live in urban poor settings may find that the private facilities meet their needs.

Women in this study preferred private providers for confidentiality and respectful treatment, which is consistent with studies about choice of private providers in Kenya [4, 15]. One qualitative study in Kenya showed that respectful care in private maternity hospitals allowed clients to form long-term relationships with providers and led women to choose these facilities over public facilities [10]. Convenience, confidentiality and respectful attitudes at private facilities may lead to higher satisfaction rates in clients who receive family planning services at private facilities.

The study findings also showed that women balanced the advantages of private facilities with their concern about low-quality technical medical treatment. Surveys of private facilities in developing countries have documented ubiquitous non-standardized medical practices [18, 19]. Women recognized that private facilities may not adhere to appropriate standards and were more likely to provide expired medications. Women acknowledged that the lack of counseling and screening at private facilities made a public facility the better choice when they wanted support for an initial decision about contraception. These results suggested that women appreciated more counseling and qualified healthcare providers, but did not *expect* to receive these services at private facilities. Explorations of patient satisfaction with medical visits show that unmet expectations, especially for counseling, lead to dissatisfaction with care [20]. High rates of satisfaction with private facilities may indicate that clients receive what they expect, quick and respectful care, and may not expect high quality medical treatment or counseling .

In contrast, public facilities were regarded as institutions with the highest quality medical care, especially for contraceptive services. Studies about quality of technical medical care show that providers in public facilities adhere to protocols and medical guidelines more often than those in private facilities [9, 16]. Women praised the public facilities for providing comprehensive information and screening measures, and chose these facilities when they needed contraceptive decision support. They believed that the counseling, screening and assessment at public facilities led to better outcomes and fewer side effects.

Despite high quality of medical treatment at public facilities, women complained about long waits and disrespectful services, which have been cited as factors that limit access to contraception in Kenya [21]. This may lead to poor satisfaction despite perceptions of high quality medical treatment. These findings bolster support for recent interventions that seek to train providers in respectful and high-quality healthcare [22].

These findings showed that women’s perception of high-quality family planning services largely revolved around ample counseling about side effects. Fear of side effects is the leading cause for non-use of contraception in Kenya [23]. Strong interpersonal relationships, created through counseling and individual provider-client interaction, contribute to quality of care [24, 25]. Poor pre-counseling about side effects is the leading cause for discontinuation and dissatisfaction with family planning methods [25, 26]. These results suggested that some women judged quality based on how they perceived the screening, counseling, and management of side effects. Those who found excellent counseling became loyal clients of that facility.

## Limitations

Women were recruited within a healthcare network served by Mathare North Health Center. Participants lived close to a public facility. Many poor urban women may not have such access to a public facility.

Furthermore, we did not differentiate between the type of private facility that the interviewee had attended (pharmacist, private hospital or NGO). Various settings may have different levels of counseling, support, standardization of medical procedures and availability of medications. Each type of private facility may need a different intervention according to its strengths and weaknesses.

Qualitative research is always subject to social desirability bias. The interviews took place in healthcare facilities, so the facilitators may have been viewed as authority figures in a government facility. Women may have responded with answers that were desirable to healthcare workers, in regards to quality of care at public facilities.

## Conclusions

Private sector health providers make up a large part of the healthcare landscape in poor urban neighborhoods. Our results suggested that private facilities will continue to attract clients by maximizing satisfaction through respectful care and efficient of service, but need assistance with technical standards of care. Public facilities could attract more clients by improving interpersonal relationships and efficiency

Quality improvement for both public and private facilities that provide family should focus on standardizing individualized counseling and side effect management. Standardization and public-private partnerships have shown overall improved quality of care. Standardization of side effects counseling and management could particularly improve client perception of quality at both private and public facilities. Educational and counseling interventions with individualized pharmacists and drug shops have shown some improvement in knowledge of providers, but have not shown increased client counseling [27, 28]. Some suggest that adding counseling to the pharmacy visits may counteract the benefits of quick and convenient services that create satisfaction [28].

Social franchises have been able to connect individual private providers into networks that improve consistent supply chain, standardize trainings and evaluations throughout Africa. These networks have taken the lead in improving quality of reproductive healthcare services provided by the private sector [29]. One of the oldest contraceptive social franchises, Marie Stopes, provides services within the Kenyan informal settlements, but these results suggest that many private facilities have not benefitted from social networking.

To create better standardization, government institutions could create accreditation system. This accreditation could be given to healthcare providers who can prove standardization of medical care, and would allow clients to make informed choices about medical facilities. As growing public-private partnerships continue to strengthen systems [30, 31], standardization of care in private facilities can improve perception of quality and access to contraception services.

## Abbreviations

CPR: Contraceptive Prevalence Rate
FDG: Focus Group Discussion
IDI: In-depth Interview
KES: Kenyan Shillings
USD: United States Dollar
MNHC: Mathare North Health Center
NGO: Non-governmental organization
